# Chicago Veterinary Society

**Published:** 1899-07

**Authors:** Joseph B. Clancy

**Affiliations:** Secretary


### CHICAGO VETERINARY SOCIETY.

The regular monthly meeting was called to order on Thursday evening, April 13th, President Robertson presiding. The minutes of the previous meeting were approved. The Board of Censors reported favorably on the applications of Drs. H. Busman and A. J. Pistor, of the U. S. B. A. I., and they were duly elected.

Dr. Joseph Hughes, chairman of the Legislative Committee, reported the results of that committee's inquiry into the attitude and sentiment of the recent candidates for the mayoralty, and found that it is perfectly and absolutely useless to try and persuade the present Mayor to consider anything favorable to the veterinarians of this city.

Dr. Griiner, related his experience before the Examining Board of the City Civil Service Commission, and recited some of the questions asked him as an applicant for the office of City Police Veterinarian, a few of which are appended here: 1. What do you know about the construction of a patrol box? 2. Of a patrol wagon? He refused to answer such exasperating questions, created a little excitement, yet passed the examination with a percentage of about 76, and reported that a former patrolman received the appointment, having passed the examination with a percentage of about 99.99, and a saloon-keeper was appointed assistant with a percentage of about 86.

JOSEPH B. CLANCY,
Secretary.

*Temperature and its Relation to Soundness.*

BY DR. A. M. CASPER.

Dr. Casper: I would like to ask the members of this society what, in their opinion, the temperature of a horse has to be in order to pronounce same unsound. Should an animal with a temperature of 103°, that is green and is for sale at the yards, be rejected? That is if there are no other symptoms of disease present. Although this high temperature is a deviation from a normal point, I do not think that such an animal should be rejected. If you would ask me if that animal was absolutely sound, I would say no; but ordinarily I would pass an animal like that, advising the owner to watch him for a day or two. I would like to hear the opinion of the members on this subject.

Dr. Campbell: I do not think that I would pass a horse that has a temperature above normal.

Dr. Griiner: I bought two horses a few weeks ago. and just for curiosity took their temperature. They had an elevation of two degrees above normal. I do not think it amounts to much. These horses are all right now, and as most of the green horses have a rise in temperature we ought to be very lenient. After they got accustomed to the barn they were all right. There was a slight loss of appetite for the first day or so.

Dr. Baker: This idea of taking the temperature of horses without any symptoms that may cause suspicion that something is wrong is a new idea to me. I never think of doing such a thing unless there is something to arouse my suspicion that there is something wrong. If there is no cold or loss of appetite present I think it is superfluous. I never had the curiosity to take the temperature anyway, so that I am unable to add anything to the history of such cases; but if it is a fact that these green horses all have an elevation of temperature, without any other symptoms of disease, I think it would be impolitic to reject them on account of that. Horses brought from the country into the city are always somewhat excited, and this excitement, aggravated by the pushing, hauling, whipping, etc., that they get in the yards, is sufficient to produce an elevation in temperature. It is known as ephemeral fever. The change of food and the different surroundings are sufficient to produce some elevation of temperature, but without any other symptoms I think it would be an injustice to reject such horses.

Dr. Howe: I have often found this condition in Jersey cattle in the yards, where the slightest little change caused some rise in temperature.

Dr. Paxson: I think that the thermometer is a very important weapon and should be used to a great extent. In cases of influenza, for instance, the thermometer points to the affection before any other symptoms are apparent, so that you can check the disease before it develops. Many times horses are found apparently well that have high temperature, and next day they are found to be very sick. I think from a scientific standpoint we should not accept a horse with abnormal symptoms.

Dr. Baker: Dr. Paxson's and my ideas are not apparently on the same point. In influenza the temperature rises suddenly, sometimes in six hours. A case of temperature at 102°, with no other symptoms but possibly a little stocking of the legs, and a temperature of 106°, are two different things. I think in a case where the temperature is above 102 or 102½° it would be fair to put the animal under treatment for twenty-four hours. If it is influenza it will develop by that time; if not, it will be normal.

Dr. Campbell: Suppose you know that a horse with temperature of 102° has been in the stall and had no excitement, would you accept that horse?

Dr. Baker: I would draw the line as to the condition of the horse. Fever and influenza develop very rapidly. You see the temperature goes up then within an hour to 106°. I would not reject a horse that does not show any other suspicious symptoms but rise in temperature to 102°.

Dr. Hughes: It would be well for us to arrive at some definite temperature. A horse with a temperature of 102½° is suspicious, but 102° is not. In a green horse there is a certain amount of indigestion present, and excitement follows. This accounts also for the swollen legs we find. A horse with 103° should be rejected. I never take a horse's temperatures any more than I analyze his urine, and do not think that a horse with a temperature of 102½° should be rejected. I would like to know whether 102° is a very normal temperature in the ring—that is, take it in the morning and night. I know that the majority of these horses whose temperature I took varied between 101–102° every day.

Dr. Campbell: Did you find an animal in normal health 100° or below?

Dr. Hughes: I did.

Dr. Campbell: I find, as a rule, that my thermometer registers between 99° and 100°, and I consider him then all right. Above that I do not, and by giving him drugs his temperature always goes down to 100°. Has the size of the horse got anything to do with it?

Dr. Hughes: I do not think it would, in particular.

Dr. Baker: I have taken the temperature of a great many horses, and I have come to the conclusion that the normal temperature of the horse is about 100°. Some German authority tested as many as six hundred at a time, and found the average 99.97°.

Dr. Day: I remember in Wichita, Kan., I had a horse to treat that is now racing. This horse was suffering from throat trouble, and I was asked to examine him. I found his temperature to be 101°. I told the owner that I did not think the horse was suitable to race, but that he could try it, as it would not hurt the horse. After the race I saw the horse, and found his temperature to be $105\frac{3}{5}$°, and I made up my mind that he did something very harmful; but, to my surprise, next morning I found that the horse's temperature was but little above 100°. I found all horses that I examined that had a temperature of little above 100° or 101°, especially race-horses, after the exertion had a temperature of as high as 104–105°, but were all right again after a little rest. If we would reject horses that had no other trouble present but high temperature we would have to reject all these horses.

Dr Robertson: I had considerable experience with shippers that go to buy and sell horses in the yards. I have had them take horses out of my hands that had a temperature of 105° and coughed. They took them into the yards and sold them as sound. If the party that purchased such a horse would have taken his temperature after going out of the ring he would have necessarily detected that there was something wrong, and I have no doubt that passing so many horses through the ring without taking their temperature has, to a certain extent, caused a prejudice against such horses, as they often get sick very shortly after they are bought and taken to the barns. I knew of a hundred that passed through the ring that way. On the other hand, I have taken the temperature of horses after a trip of four to five miles and found their temperature to be from 104–5°. I recommend all such horses to stay in the barn till the next morning, and, as a rule, find their temperature to be normal. The excitement of the trip raised the temperature. If the temperature did not go down next morning I rejected the horse. Last week I bought a horse in the yards with a temperature of 102°. I led him down behind a buggy, put him into the barn, took his temperature, and found it to be 104°. I left him in the barn till next morning, took his temperature then, and found it to be 101°. In heavy horses, as a rule, I find that their normal temperature is between 101–102°. My experience with heavy horses is, that if they quit eating they are very seriously sick. If this is the case, even if their temperature is but 102°, we ought to commence treating them at once, as we can thus prevent serious illness. Some barns make it a rule to give all new horses that just come into the barn a little fever medicine. I think it is a good idea to take the temperature of all such horses that come from the yards.

Dr. Paxson: I would like the Society to come to a conclusion as to what would be the highest temperature with which we could allow a horse to pass.

Dr. Robertson: I think $102\frac{1}{2}$°

Dr. Hughes: What is the normal temperature of the horse? I think 101°.

Dr. Robertson: My experience is largely with these heavy draught-horses. There were some whose temperatures I took every morning, and found that it was very close to 102°. I presume it was because, as a rule, they were in close confinement and had but little exercise. I always supposed that the normal temperature was 100°. If anyone would ask me what the normal temperature of a horse is, I would say in heavy horses from 101–102°, in light horses about 100°. I never bother my head if the temperature of a heavy horse is 102°, and I pronounce him sound. In light horses I have not had so much experience, as I have not taken their temperature so frequently.

100° I should judge, would be considered the normal temperature of such horses.

Dr. Hughes: What is the normal pulse of a horse?

Dr. Pister: I find that heavy Percheron and Normandy horses in this section of the country have a pulse of about 30–32—that is, heavy stallions. The pulse of all these horses varies very much. It varies from 3 to 5 beats. It makes a difference between country and city horses. The former are not apt to get so excited, hence their pulse is much lower.

Dr. Baker: I find, as an average, a horse's pulse is about 35–36. I have found many cases as low as 25–28 in draught-horses.

Dr. Pister: I found heavy draught-horses in New York whose pulse varies between 36–45. I should suppose that anything between 36–45, where there are no symptoms of any other trouble present, should be considered normal. It is a hard thing, however, to set down any rule that would be actually a normal pulse.

Dr. Hughes: I think that the normal pulse is between 40–42.

